# In vivo monitoring of active subretinal fibrosis in mice using collagen hybridizing peptides

**DOI:** 10.1038/s41684-024-01408-0

**Published:** 2024-07-26

**Authors:** Markus Linder, Lucas Bennink, Richard H. Foxton, Mike Kirkness, Peter D. Westenskow

**Affiliations:** 1 Roche Pharma Research and Early Development, Roche Innovation Center, F. Hoffmann-La Roche AG, Basel, Switzerland; 2 3Helix, Salt Lake City, UT USA; 3 Present Address: Novartis Institutes for BioMedical Research, Basel, Switzerland

**Keywords:** Mechanisms of disease, Optical imaging, Animal disease models

## Abstract

Subretinal fibrosis is associated with worse visual outcomes in patients with neovascular age-related macular degeneration. As there is a lack of optimal biomarkers and no method that directly detects collagen in the back of the eye, novel tools that monitor fibrosis-related changes in neovascular age-related macular degeneration are needed. Here, using two mouse models (the laser-induced choroidal neovascularization model, and the JR5558 mouse presenting with spontaneous subretinal neovascularization with fibrosis), we imaged active fibrotic lesions using fluorescently labeled collagen hybridizing peptides (CHPs), short peptides that bind to single α-chain collagen structures during collagen remodeling. JR5558 retinal pigment epithelium/choroid flat mounts showed CHP co-staining with fibrosis and epithelial mesenchymal transition-related markers; additionally, CHP histopathology staining correlated with in vivo CHP imaging. After laser-induced choroidal neovascularization, in vivo CHP binding correlated with laser intensity, histopathology CHP and fibronectin staining. Laser-induced choroidal neovascularization showed decreased CHP intensity over time in healing/regressing versus active scars in vivo, whereas increased CHP binding correlated with elevated fibrosis in JR5558 mouse eyes with age. In bispecific angiopoietin 2/vascular endothelial growth factor antibody-treated JR5558 mice, CHPs detected significantly decreased collagen remodeling versus immunoglobulin G control. These results demonstrate the first use of CHPs to directly image remodeling collagen in the eye and as a potential clinical optical biomarker of active subretinal fibrosis associated with ocular neovascularization.

## Abstract (web summary)

This study reports the use of fluorescently labeled collagen hybridizing peptides to directly image collagen remodeling and monitor fibrosis in two mouse models of ocular neovascularization.

## Main

Neovascular age-related macular degeneration (nAMD) is a leading cause of blindness and visual impairment worldwide^1,2^ and is characterized by neovascularization in the back of the eye, including the choroid and retina. In nAMD, the newly formed vascularization is associated with the upregulation of multiple cytokines and growth factors, including vascular endothelial growth factor (VEGF) and angiopoietin 2 (Ang-2), that promote vascular instability, contributing to vascular leakage, neovascularization and inflammation^3,4^. If not regulated, chronic inflammation, uncontrolled wound healing and subretinal fibrosis can be triggered, which results in the excessive deposition of extracellular matrix proteins, such as collagen and fibronectin within the subretinal space^5–10^. Despite treatment with anti-VEGF therapies such as aflibercept, ranibizumab or bevacizumab, the current gold standard of patient care for nAMD, approximately half of patients with nAMD can develop subretinal fibrosis^6,11^. The presence of fibrotic scarring is strongly associated with worse visual outcomes in eyes with nAMD, even after treatment with anti-VEGF therapies^11–15^. Furthermore, nAMD with subretinal fibrosis is the leading cause of blindness in the elderly population of developed nations^9^. In addition, the formation of fibrotic tissue is an important contributor to retinal detachment in proliferative diabetic retinopathy^16^. Despite this, there are limited treatment options for patients with subretinal fibrosis, with no US Food and Drug Administration-approved therapeutics currently available^9^.

The ability to identify and monitor subretinal fibrotic activity during disease progression or treatment of nAMD are key challenges in the development of therapies that target or prevent subretinal fibrosis^17,18^. Collagen remodeling and overall collagen concentration within the fibrotic lesion is modulated primarily by fibroblast cells, which produce both collagen and matrix metalloproteinases (a class of enzymes responsible for collagen cleavage) in response to environmental cues, including many from collagen itself^19,20^. Collagen fragments, produced during collagen remodeling, can act as cell signaling peptides to modulate inflammatory pathways and collagen production^21,22^. During active fibrotic disease and progression, there is an increase in collagen production and degradation, and previous findings suggest collagen fragments as a potential biomarker for fibrotic diseases such as nonalcoholic steatohepatitis^23–26^, idiopathic pulmonary fibrosis^27–30^ and other fibrotic diseases^31–34^. Taken together, these findings suggest that active fibrosis can be considered a disease of increased collagen remodeling and overall collagen deposition within the fibrotic lesion. The leading diagnostic tool for retinal fibrosis in the clinic today is optical coherence tomography (OCT), a noninvasive tool that images the cross-sectional area of the retina and vascularization^35^. Subretinal hyperreflective material (SHRM) is considered the standard OCT readout for fibrosis in nAMD; however, because the composition can include collagen, drusen, fibronectin, elastin, fat or other extracellular matrix proteins, SHRM cannot differentiate collagen from these other materials^15,36,37^. This highlights the need for a complementary approach that can specifically diagnose and monitor the presence of remodeling collagen.

Collagen hybridizing peptides (CHPs) are short peptide sequences that bind to denatured/remodeling collagen via a triple helix-forming mechanism^38–42^. Since their binding mechanism is based on structural recognition of individual α-chains that are exposed during remodeling, CHPs have high affinity for denatured collagen and no affinity toward healthy, fully folded (triple-helical) collagen molecules^38–42^. As all collagen subtypes contain triple-helical regions and because collagen is conserved across species, CHPs are not species specific and can bind to all 28 types of collagen, enabling direct detection of total collagen turnover as a single probe^39,42^. In addition, due to their neutral, hydrophilic and proline-rich sequence, CHPs exhibit low nonspecific binding and maintain high serum stability, enabling systemic delivery via tail vein injections in mice^43^. CHPs have been used as a probe for detecting increased collagen remodeling in many fibrotic disease states, including kidney and pulmonary fibrosis in animal models^39^, and have predicted fibrotic outcomes in liver biopsy samples from patients with biliary atresia^24^.

In this study, we aimed to identify and monitor subretinal fibrosis caused by ocular neovascularization in vivo using fluorescently labeled CHPs to visualize remodeling collagen in two mouse models of subretinal neovascularization with fibrosis: the laser-induced choroidal neovascularization (LCNV) model and the JR5558 mouse. LCNV is a well-established mouse model of acute choroidal neovascularization (CNV) lesions, which follows predictable stages of development similar to CNV in humans^44,45^. JR5558 mice spontaneously develop neovascular lesions that invade the subretinal space; this model can be used to study early and late events associated with subretinal neovascularization with fibrosis, is validated for drug discovery and has been used for investigating therapeutic targets in nAMD^4,46–49^.

## Results

### R-CHPs bind specifically to remodeling collagen of fibrotic tissue from subretinal ocular neovascularization lesions

**Fig. 1:**
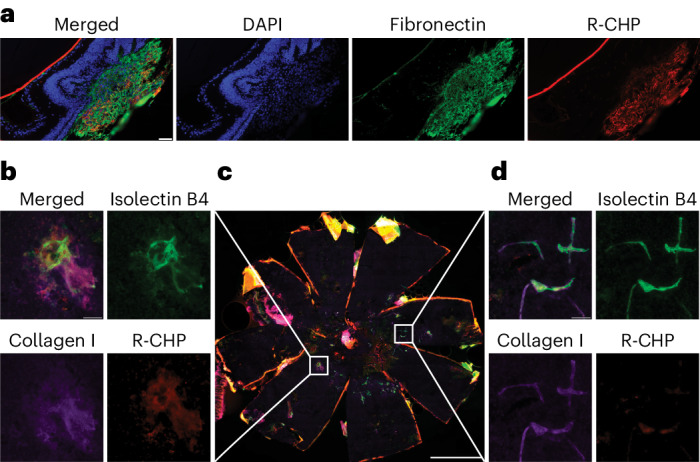
IHC of R-CHP binding in LCNV and JR5558 flat mounts. **a**, CNV was induced in C57BL/6 mouse eyes with a 532α green laser (300 mW). Representative images 14 days after laser injury of RPE/choroid flat mounts from LCNV mice stained for DNA (DAPI), fibronectin and R-CHP binding. **b**–**d**, Representative images of RPE/choroid flat mounts from 50-day-old JR5558 mice showing areas of fibrosis (**b**) and healthy tissue (**d**) stained for isolectin B4 (a vascular marker), collagen I and R-CHP binding. Highlighted regions (**c**) correspond to a healthy area (right) and a fibrotic lesion (left). Scale bars, 1 mm (**c**) and 50 µm (**a**, **b** and **d**).

To evaluate CHP-binding specificity for remodeling collagen associated with ocular neovascularization, retinal pigment epithelium (RPE)/choroid flat mounts from LCNV and JR5558 mouse eyes were stained with sCy3-labeled CHPs (R-CHPs; used exclusively as the ex vivo probe in this study) and binding was assessed using immunofluorescence (Fig. 1). Overall, in LCNV flat mounts 14 days after laser injury, R-CHPs bound in a similar location to fibronectin staining, a commonly used biomarker for subretinal fibrosis, indicating an association of R-CHP binding with fibrosis (Fig. 1a). In fibrotic lesions of 50-day-old JR5558 flat mounts, R-CHPs bound in a similar location to collagen I and isolectin B4 staining on and surrounding the vessels (Fig. 1b,c, highlighted region on the left). By contrast, the staining overlap of R-CHP with collagen I and isolectin B4 was greatly reduced in healthy (intact collagen) areas of JR5558 flat mounts where collagen deposition was limited to the blood vessels only (Fig. 1c, highlighted region on the right, and Fig. 1d). These results indicate that CHPs are specific for denatured collagen, which is upregulated in active fibrotic lesions, while having minimal affinity for collagen that is not undergoing remodeling, such as that surrounding quiescent vasculature.

**Fig. 2:**
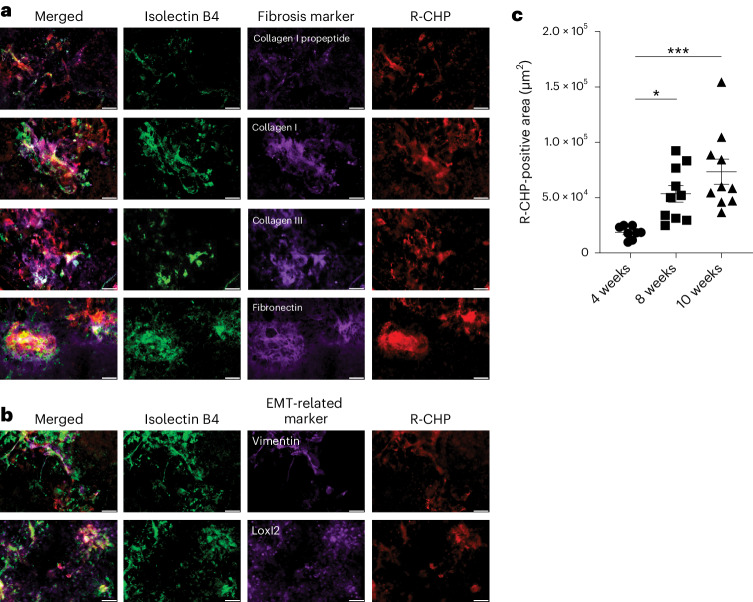
Fluorescent IHC of R-CHP binding and fibrosis and EMT-related markers in JR5558 RPE/choroid flat mounts. **a**,**b**, Representative images of RPE/choroid flat mounts from 70-day-old JR5558 mice stained for R-CHP binding and fibrosis (collagen I propeptide, collagen I, collagen III and fibronectin) (**a**) and EMT-related (vimentin and lysyl oxidase-like 2 (Loxl2)) (**b**) biomarkers. **c**, R-CHP-positive area in RPE/choroid flat mounts from 28-, 56- and 70-day-old JR5558 mice (28 days, *n* = 8; 56 and 70 days, *n* = 10). The data in **c** are mean (±s.e.m.). Ordinary one-way ANOVA with Tukey’s multiple comparisons test in **c**; *P < 0.05, ***P < 0.001. Scale bars, 50 µm.

In 70-day-old JR5558 flat mounts, R-CHPs bound in a similar location to fibrosis (fibronectin, collagen I propeptide and collagen I and III) (Fig. 2a) and endothelial-to-mesenchymal transition (EMT)-related markers (vimentin and lysyl oxidase-like 2) (Fig. 2b). The relative area of collagen remodeling as measured by R-CHP binding (mean ± standard error of the mean (s.e.m.)) in JR5558 RPE/choroid increased significantly in 56-day-old (53,467 µm^2^ ± 7,647) and 70-day-old (73,355 µm^2^ ± 11,273) mice compared with 28-day-old mice (18,600 µm^2^ ± 2,029) (*P* < 0.05 and *P* < 0.001, respectively; ordinary one-way analysis of variance (ANOVA) with Tukey’s multiple comparisons test), demonstrating that R-CHPs bind increased collagen remodeling associated with increased fibrosis as JR5558 mice age (Fig. 2c).

### sCy7.5-CHPs bind to remodeling collagen within JR5558 subretinal neovascularization lesions in vivo

To enable in vivo imaging of collagen in subretinal fibrosis, CHPs were labeled with the fluorophore sulfo-cyanine 7.5 (sCy7.5; excitation/emission: 778/797 nm), which has a similar excitation/emission profile to indocyanine green (excitation/emission: 788/813 nm) and can be imaged using a scanning laser ophthalmoscope in the same manner as indocyanine green angiography, a common diagnostic method for assessing nAMD (Supplementary Fig. 1). To facilitate in vivo imaging, a sCy7.5 fluorophore was conjugated to a (GfO)_9_-CHP because this CHP design resists self-trimerization and does not require preheating^38^. Targeted and control sCy7.5-CHPs were synthesized with a sequence that hybridizes with damaged collagen (targeted sCy7.5-CHP) or a scrambled sequence, which removes from the CHP the prototypical Gly–X–Y sequence found in collagen, as a control (nontargeted sCy7.5-CHP, which does not bind damaged collagen). The control sequence contains the same amino acid composition and sCy7.5 fluorophore; therefore, the molecular weight and overall charge were identical to the targeted sCy7.5-CHP. In fibrotic lesions of RPE/choroid flat mounts from 84-day-old JR5558 mice, binding of targeted sCy7.5-CHPs, but not control sCy7.5-CHPs, co-localized with R-CHP binding, demonstrating that sCy7.5-CHPs bind specifically to remodeling collagen and can be used to image fibrosis (Supplementary Fig. 2).

**Fig. 3:**
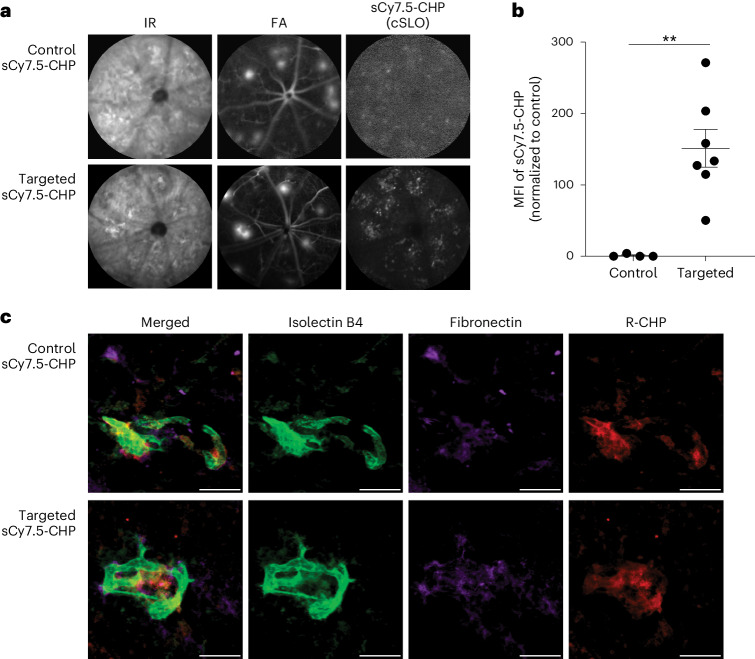
In vivo imaging of sCy7.5-CHP binding to remodeling collagen in JR5558 mice. Seventy-day-old JR5558 mice received targeted or control sCy7.5-CHPs i.v. and were analyzed 5 days later using in vivo imaging followed by ex vivo IHC. **a**, Representative images of JR5558 retinas showing IR, FA and sCy7.5-CHP in vivo binding. **b**, MFI of control and targeted sCy7.5-CHPs in JR5558 retinas (control, *n* = 4; targeted, *n* = 7). **c**, Representative images of RPE/choroid flat mounts from JR5558 mice (previously injected with sCy7.5-CHPs) co-stained for R-CHP binding, isolectin B4 and fibronectin. The data in **b** are mean (±s.e.m.) normalized to control. Unpaired *t*-test with Welch’s correction in **b**; **P < 0.01. Images in **a** cover 1 mm of the retina. Scale bars 50 µm.

To evaluate the capacity of sCy7.5-CHPs to image fibrosis in vivo, 70-day-old JR5558 mice were injected intravenously (i.v.) with targeted or control sCy7.5-CHPs and assessed 5 days later using infrared reflectance (IR), fluorescein angiography (FA) and confocal scanning laser ophthalmoscopy (cSLO). Targeted, but not control, sCy7.5-CHPs were detected with cSLO (Fig. 3a). Mean fluorescence intensity (MFI) (mean ± s.e.m.) of sCy7.5-CHPs increased significantly with targeted sCy7.5-CHPs (56,131 ± 8,125) versus control sCy7.5-CHPs (6,031 ± 2,920) (*P* < 0.01 (unpaired *t*-test with Welch’s correction); Fig. 3b). Ex vivo staining with isolectin B4, fibronectin and R-CHP after the in vivo imaging confirmed the presence of fibrosis and damaged collagen in the control and targeted CHP JR5558 mouse groups. Thus, the lack of cSLO signal observed was due to the inability of the control sCy7.5-CHPs to bind remodeling collagen (Fig. 3c) and not because of a lack of denatured or remodeling collagen. Taken together, these results demonstrate that sCy7.5-CHPs bound directly to, and enabled in vivo imaging of, collagen in subretinal fibrosis.

### sCy7.5-CHP binding to remodeling collagen enables quantification of active fibrosis after LCNV in vivo

**Fig. 4:**
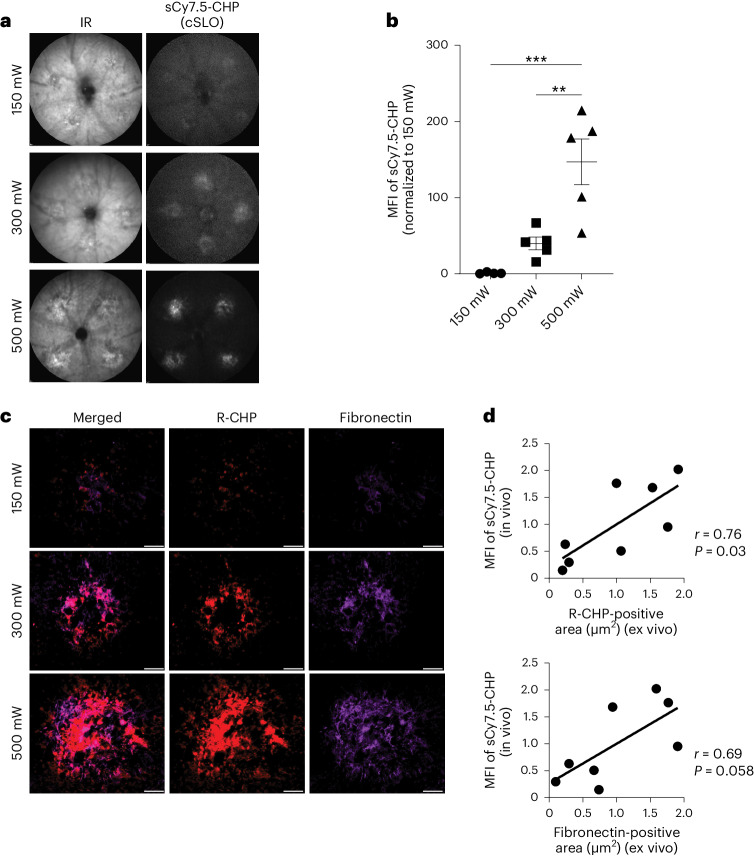
In vivo imaging of sCy7.5-CHP binding to remodeling collagen in LCNV mice. CNV was induced in C57BL/6 mouse eyes with a 532α green laser; increasing laser intensities (150, 300 and 500 mW) were used to produce increasing severities of retinal fibrosis. After 9 days, LCNV mice received sCy7.5-CHPs i.v. and were analyzed 5 days later using in vivo imaging followed by ex vivo fluorescent IHC. **a**, Representative images of LCNV mouse retinas showing IR and sCy7.5-CHP in vivo binding. **b**, MFI of sCy7.5-CHPs in LCNV retinas (150 mW, *n* = 4; 300 mW and 500 mW, *n* = 5). **c**, Representative images of RPE/choroid flat mounts from LCNV mice (previously injected with sCy7.5-CHPs) co-stained for R-CHP binding and fibronectin. **d**, Correlation between MFI of sCy7.5-CHPs versus ex vivo R-CHP-positive area (normalized to the average) (top; *n* = 8), and versus ex vivo fibronectin-positive area (normalized to the average) (bottom; *n* = 8). The data in **b** are mean (±s.e.m.) normalized to 150 mW. Ordinary one-way ANOVA with Tukey’s multiple comparisons test in **b**; simple linear regression and two-tailed correlation with Pearson correlation coefficients *r* and *P* in **d**; **P < 0.01, ***P < 0.001. Images in **a** cover 1 mm of the retina. Scale bars, 50 µm.

To assess the sensitivity of CHPs to bind to and enable in vivo imaging of remodeling collagen in fibrotic lesions of differing severity, in vivo imaging with sCy7.5-CHPs was performed using the LCNV mouse model. Laser intensities were varied from 150 to 500 mW to induce advancing severities of laser damage and subsequent fibrosis in LCNV mouse eyes. Mice were injected with sCy7.5-CHPs 9 days later, when collagen remodeling is high and ongoing, and IR and cSLO were performed 5 days later to evaluate CHP binding (Fig. 4a). sCy7.5-CHP binding correlated with increasing laser intensity and, thus, increasing fibrosis severity. The MFI (mean ± s.e.m.) of sCy7.5-CHP increased significantly with 500 mW (517,514 ± 105,565) compared with 300 mW (140,288 ± 29,647; *P* < 0.01) and 150 mW (3,520 ± 1,863; *P* < 0.001 (ordinary one-way ANOVA with Tukey’s multiple comparisons test); Fig. 4b). Fibrosis severity was confirmed ex vivo with R-CHP and fibronectin staining (Fig. 4c). There was a correlation between sCy7.5-CHP binding in vivo and R-CHP binding ex vivo (*r* = 0.76, *P* = 0.03) and a trend for a correlation between sCy7.5-CHP binding in vivo and fibronectin binding ex vivo (*r* = 0.69, *P* = 0.058 (simple linear regression and two-tailed correlation with Pearson correlation coefficients); Fig. 4d).

**Fig. 5:**
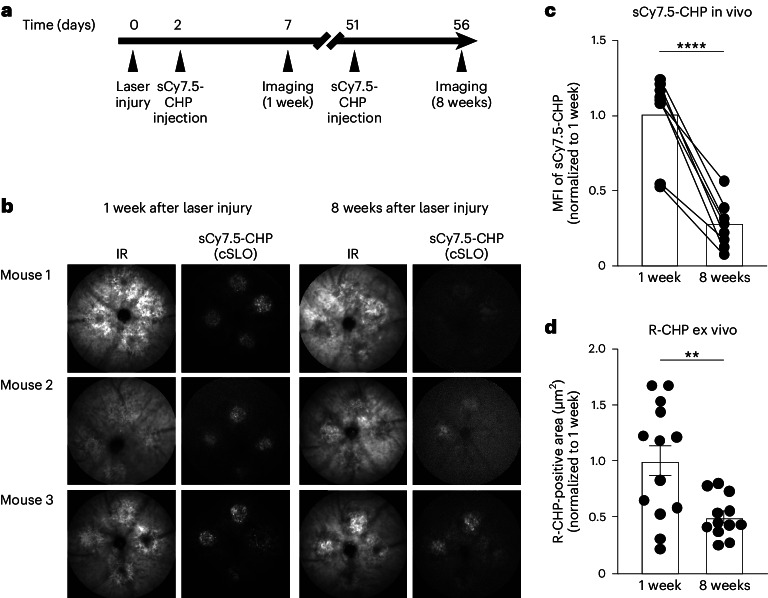
In vivo imaging of sCy7.5-CHP binding to remodeling collagen in LCNV mice over time. **a**, Schematic of experiment showing timing of 532α green laser (300 mW) injury to C57BL/6 mouse eyes, sCy7.5-CHP intravenous injection and imaging. **b**, Representative images of LCNV mouse retinas showing IR and sCy7.5-CHP in vivo binding. **c**, MFI of sCy7.5-CHPs in LCNV mouse retinas (*n* = 8 per time point). **d**, R-CHP-positive area in RPE/choroid flat mounts from LCNV mice (1 week, *n* = 13; 8 weeks, *n* = 12). The data in **c** are mean normalized to 1 week; the data in **d** are mean (±s.e.m.) normalized to 1 week. Paired *t*-test in **c**; unpaired *t*-test with Welch’s correction in **d**; **P < 0.01, ****P < 0.0001. Images in **b** cover 1 mm of the retina.

To evaluate the capacity of sCy7.5-CHPs to image decreased collagen remodeling in healing versus fresh wounds in vivo, LCNV mouse eyes were imaged with IR and sCy7.5-CHPs 1 and 8 weeks after laser injury (Fig. 5a). sCy7.5-CHP binding was decreased in healing scars compared with fresh wounds (Fig. 5b), evidenced by a significant reduction in the MFI of sCy7.5-CHP at 8 weeks (18,188 ± 3,918) compared with 1 week after laser injury (69,576 ± 7,187; *P* < 0.0001 (paired *t*-test); Fig. 5c). Ex vivo R-CHP staining confirmed reduced collagen turnover through the 8-week healing process after the initial injury to the LCNV mouse eyes. The area of R-CHP binding (mean ± s.e.m.) significantly decreased from 19,605 µm^2^ ± 2,787 at 1 week to 9,775 µm^2^ ± 1,047 at 8 weeks (*P* < 0.01 (unpaired *t*-test with Welch’s correction); Fig. 5d).

Taken together, these LCNV data sets indicate that sCy7.5-CHP binding correlated with fibrosis severity and enabled imaging of reduced collagen remodeling over time in laser-induced scarring.

### sCy7.5-CHPs enabled therapeutic efficacy monitoring of bispecific anti-Ang-2/VEGF antibody treatment in JR5558 mice

**Fig. 6:**
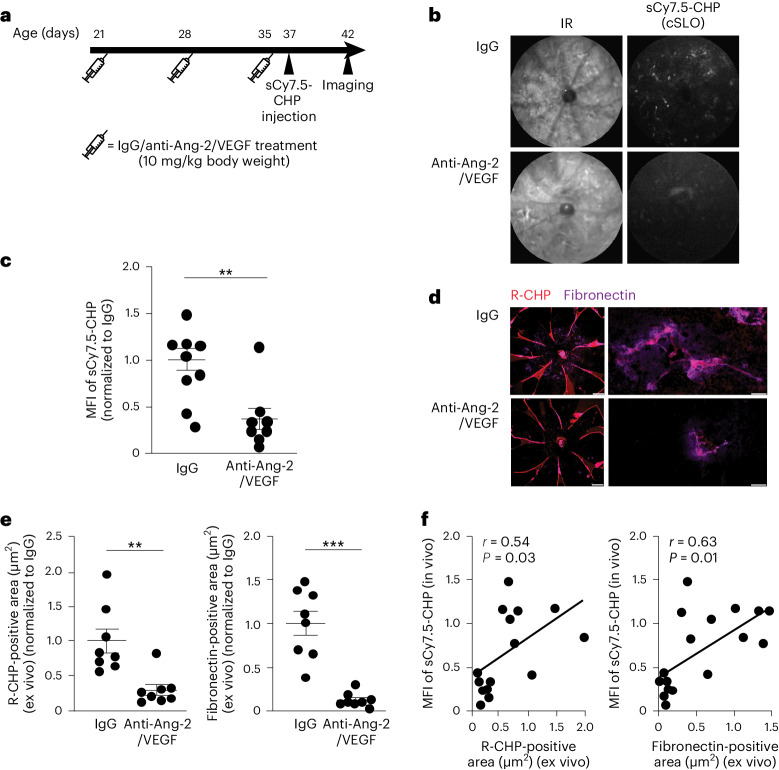
In vivo imaging of sCy7.5-CHP binding to remodeling collagen following anti-Ang-2/VEGF-A antibody treatment of JR5558 mice. **a**, Schematic of experiment showing timing of IgG or anti-Ang-2/VEGF antibody (10 mg/kg) injection, sCy7.5-CHP intravenous injection and imaging of JR5558 mice. **b**, Representative images of JR5558 mouse retinas showing IR and sCy7.5-CHP in vivo binding. **c**, MFI of sCy7.5-CHPs in JR5558 retinas (*n* = 8 per treatment). **d**, Representative images of RPE/choroid flat mounts from JR5558 mice (previously injected with sCy7.5-CHPs) treated with IgG or anti-Ang-2/VEGF antibody and co-stained for R-CHP binding and fibronectin. The highlighted regions correspond to fibrotic lesions. **e**, R-CHP-positive area (left) and fibronectin-positive area (right) in RPE/choroid flat mounts from JR5558 mice (*n* = 8 per treatment). **f**, Correlation between MFI of sCy7.5-CHPs versus ex vivo R-CHP-positive area (normalized to the average; left), and versus fibronectin-positive area (normalized to IgG; right) (*n* = 16 for both panels). The data in **c** and **e** are mean (±s.e.m.) normalized to IgG. Unpaired *t*-test in **c** and **e**, left; unpaired *t*-test with Welch’s correction in **e**, right; simple linear regression and two-tailed correlation with Pearson correlation coefficients *r* and *P* in **f**; **P < 0.01, ***P < 0.001. Images in **b** cover 1 mm of the retina. Scale bars, 1 mm (**d**, left) and 50 µm (**d**, right).

To assess the capacity of sCy7.5-CHPs to monitor the fibrotic prevention of a bispecific anti-Ang-2/VEGF antibody in vivo, sCy7.5-CHP binding was imaged in 42-day-old JR5558 mice after three-weekly anti-Ang-2/VEGF antibody or IgG control injections (Fig. 6a). sCy7.5-CHP binding in vivo was reduced in anti-Ang-2/VEGF antibody-treated mice compared with the IgG control (Fig. 6b). The MFI (mean ± s.e.m.) of sCy7.5-CHP significantly decreased from 46,914 ± 5,358 with the IgG control to 17,093 ± 5,499 with anti-Ang-2/VEGF antibody treatment (*P* < 0.01 (unpaired *t*-test); Fig. 6c). Decreased fibrosis with anti-Ang-2/VEGF antibody treatment versus IgG control was confirmed by ex vivo staining for denatured collagen (R-CHP) and fibronectin (Fig. 6d). The R-CHP-positive area (mean ± s.e.m.) significantly decreased from 23,064 µm^2^ ± 3,986 with the IgG control to 6,554 µm^2^ ± 1,842 with anti-Ang-2/VEGF antibody treatment (*P* < 0.01 (unpaired *t*-test); Fig. 6e, left), and the fibronectin-positive area significantly decreased from 200,649 µm^2^ ± 28,021 with IgG control to 24,379 µm^2^ ± 5,870 with anti-Ang-2/VEGF antibody treatment (*P* < 0.001 (unpaired *t*-test with Welch’s correction); Fig. 6e, right). There was a correlation between the sCy7.5-CHP binding in vivo and R-CHP binding ex vivo (*r* = 0.54, *P* = 0.03 (simple linear regression and two-tailed correlation with Pearson correlation coefficients); Fig. 6f, left) and between sCy7.5-CHP binding in vivo and fibronectin binding ex vivo (*r* = 0.63, *P* = 0.01 (simple linear regression and two-tailed correlation with Pearson correlation coefficients); Fig. 6f, right).

These results indicate that sCy7.5-CHP binding in vivo enables monitoring of the fibrotic prevention effects of dual Ang-2/VEGF-A inhibition in the retina.

## Discussion

In this study, we report the use of fluorescently labeled CHPs to identify and visualize remodeling collagen in active fibrotic lesions in the eye and for the monitoring of subretinal fibrosis progression associated with nAMD in two mouse models of ocular neovascularization. Furthermore, we demonstrated the apparent antifibrotic effects of a bispecific anti-VEGF/Ang-2 antibody, highlighting the potential for CHPs to support clinical development of nAMD treatments and antifibrotic therapeutics.

Recently published findings have identified collagen degradation fragments as novel biomarkers for monitoring fibrotic progression; however, in these reports, antibodies were used for ex vivo examination using serum^25,29,50^. Current antibody designs are limited by their recognition of specific epitopes, which may be destroyed by enzymatic degradation during remodeling processes, and a high potential for nonspecific binding; therefore, antibodies cannot be easily used for in vivo diagnostic or imaging purposes. Due to the ubiquitous nature and the highly repetitive sequence of collagen, raising monoclonal antibodies to denatured collagen is difficult. Consequently, nonspecific and off-target binding is a common issue with collagen monoclonal antibodies raised to collagen fragments as the denatured collagen fragments contain the same sequences as healthy, intact (triple-helical) collagen. CHPs address both of these issues based on their structural recognition of the collagen α-chains present during collagen turnover, allowing for reliable targeting and binding of fragments from any collagen subtype without concern of ‘target site’ loss while maintaining negligible binding to healthy intact collagen^38,39,41^. Structural recognition of the collagen α-chains enables CHPs to directly image total collagen turnover as a single probe, which is important because of the various subtypes of collagen found throughout the eye. For example, CHPs would be expected to directly image turnover of collagen subtypes I, III and IV in the Bruch’s membrane during nAMD progression^6,9,51^.

In the present study, R-CHP binding of the RPE/choroid ex vivo was in a similar location to fibrosis and EMT-related markers, and the staining overlap was increased in active fibrotic lesions containing higher concentrations of molecularly damaged collagen compared with areas of healthy tissues where collagen is predominately found in triple-helical form. In addition, ex vivo staining with R-CHPs detected increased collagen remodeling in JR5558 mouse eyes as they aged, which is expected given that the fibrosis in JR5558 mice is progressive^52^. Because CHPs are unique in binding specifically to remodeling collagen, R-CHP staining did not always overlap completely with the fibrosis and EMT-related markers. We also observed some overlap of R-CHP binding with isolectin B4 staining. The vasculature contains collagen and shows a basal level of collagen remodeling, which allows CHPs to bind and show some overlap with isolectin B4 staining. Overall, these results strongly suggest that CHPs specifically bind to and identify remodeling collagen in fibrotic lesions associated with subretinal neovascularization and can be considered an improved probe for remodeling collagen in subretinal fibrosis over traditional antibody-/immunohistochemistry (IHC)-based staining techniques. CHPs can also be used in conjunction with traditional techniques ex vivo.

Subretinal fibrosis is a substantial factor in the continued loss of vision over time experienced by patients with nAMD^53,54^. Approximately half of patients with nAMD develop subretinal fibrosis despite receiving treatment with anti-VEGF therapies^6,11^; therefore, new therapies targeting additional pathways that prevent or reduce fibrosis are critical. Despite consideration of SHRM as a diagnostic readout of fibrosis in nAMD^37^, the molecular composition and specificity of this imaging biomarker for detecting fibrosis is currently unknown. These observations highlight the unmet need for direct detection of subretinal fibrosis to enable earlier diagnosis and improve monitoring of fibrosis progression as well as predictions of fibrosis outcomes, and in the discovery and development of novel therapies. Live animal imaging with CHPs for the monitoring of fibrotic diseases has shown diagnostic potential in various organ systems; however, using CHPs in vivo for specifically monitoring subretinal fibrosis has been unexplored until now^38,41,55^.

By using cSLO to detect binding of sCy7.5-CHPs in JR5558 and LCNV mouse retinas, we were able to detect and image active remodeling collagen in subretinal fibrosis in vivo for the first time. The excitation/emission profile of sCy7.5 is similar to that of indocyanine green, whose near-infrared peak emission allows deeper penetration of the retina, which is ideal for imaging subretinal layers^56^. In LCNV, sCy7.5-CHP binding in vivo correlated with R-CHP binding but not fibronectin staining, ex vivo. Direct correlation between fibronectin staining and CHPs would not be expected because they are biomarkers for similar but different disease characteristics. Fibronectin represents a secondary marker of fibrosis stage, whereas CHP binding is a direct biomarker for active fibrosis (remodeling collagen). In laser-induced scarring, in vivo binding of sCy7.5-CHP increased with increasing fibrosis (laser intensity) and could detect increased remodeling in fresh wounds versus healing scars. These important observations link collagen turnover to subretinal fibrosis progression diagnostically, thereby identifying remodeling collagen as a novel biomarker for monitoring fibrotic progression associated with nAMD in vivo. This method has the potential to support clinical development of nAMD treatments targeting fibrosis by allowing longitudinal assessments of fibrotic progression over time. Indeed, following anti-Ang-2/VEGF antibody treatment of JR5558 mice, imaging with cSLO detected reduced binding of sCy7.5-CHPs versus IgG, suggesting that the bispecific anti-Ang-2/VEGF antibody decreased fibrosis in JR5558 mouse eyes, possibly through the mechanism of enhanced vascular stabilization. Further potential advantages of in vivo CHP imaging are the use of repeated measures, which can increase statistical power, and the reduction of animal numbers in a study compared with ex vivo staining methods at multiple time points. In addition, imaging of sCy7.5-CHPs with cSLO has the potential to personalize treatment for patients with nAMD depending on the detection of active fibrosis evaluated by sCy7.5-CHP binding, for example, by identifying patients who have fibrosis that has progressed beyond the point where anti-VEGF therapy alone can be effective.

A potential limitation of the current study is applying the findings from mouse models of nAMD to humans, as both the LCNV and JR5558 mouse models have their advantages and disadvantages. LCNV in mice is a model of acute injury and inflammation that mimics a healed/healing wound; therefore, in this study, the reduced in vivo CHP binding observed at 8 weeks versus 1 week after laser injury is most likely due to healing of the laser-induced damage rather than a stabilized scar. However, we would expect similar results in a stabilized versus remodeling scar in other disease models, such as chronic kidney disease for kidney fibrosis. In the deoxycorticosterone acetate-salt angiotensin II kidney fibrosis mouse model, reduced in vivo CHP binding was also observed; however, according to other disease metrics, such as urinary albumin excretion, disease severity worsened with time and the lack of in vivo CHP binding was not due to disease resolution (unpublished data). Recently, a LCNV model has been developed that includes a second laser burn to induce CNV that transitions to a fibrovascular membrane^57^. Compared with the traditional LCNV model used here, the two-hit LCNV mouse model may mimic human nAMD more closely by inducing larger lesions that persist and remain vascularized up to 40 days and show higher levels of proinflammatory genes and higher numbers of infiltrating immune cells. Therefore, it would be of interest to analyze CHP binding in the two-hit model over time compared with the traditional LCNV model. In contrast to LCNV, the JR5558 mouse spontaneously develops neovascular lesions that are persistent and do not regress, representing a chronic model of subretinal fibrosis that allows for the study of both early and late fibrosis-associated events^48^, including the development of fibrotic changes via EMT, which reflects the progress of CNV lesions^52^. On the other hand, the JR5558 phenotype occurs in young mice and therefore may not be the ideal model to mimic the age-related human disease.

To improve the clinical utility of CHPs for the detection and monitoring of active fibrosis, future studies should evaluate the pharmacokinetics/pharmacodynamics, biodistribution and refolding kinetics of CHPs and determine the optimal route of delivery (intravenous versus intravitreal). Next-generation CHP designs can then be optimized for the best clinical utility. We performed in vivo imaging 5 days after sCy7.5-CHP injection, which is a limitation that needs to be addressed before CHPs can be used in the clinic. In addition, the potential for CHP binding to interfere in the disease process is a question that remains to be fully investigated. However, early optical density data suggest that CHP binding to denatured collagen does not interfere with collagen fibril growth or degradation, presumably due to the small area that they interact with^58^. In the clinic, it may not be possible to differentiate between fibrotic-induced remodeling versus wound healing by using CHPs alone. Therefore, other diagnostic methods, including traditional OCT and angiography, may be necessary to confirm that nAMD is present and that CHP binding is due to subretinal fibrosis. It will also be important to explore novel CHP designs that incorporate additional compounds to deliver antifibrotic drugs directly to sites of active fibrosis or to allow for new medical imaging modalities. Finally, to understand the antifibrotic effects of dual Ang-2/VEGF inhibition mechanistically, it will be important to compare with in vivo CHP imaging after anti-VEGF and anti-Ang-2 monotherapy.

In conclusion, fluorescently (near-infrared)-labeled CHPs enabled direct imaging, quantification and monitoring of collagen remodeling in vivo in fibrotic lesions associated with nAMD in two mouse models of ocular neovascularization with fibrosis. These results highlight the potential for in vivo sCy7.5-CHPs to become a key diagnostic tool for detection of active fibrosis in the retina and support the clinical development of nAMD treatments that impact fibrosis.

## Methods

### Study approval

Animal experiments were approved by the Federal Food Safety and Veterinary Office of Switzerland (reference BS‐2734) and conducted in strict adherence to the Swiss federal ordinance on animal protection and welfare, as well as according to the rules of the Association for Research in Vision and Ophthalmology Statement for the Use of Animals in Ophthalmic and Vision Research guidelines, European Directive 86/609/EEC and the Roche Ethics Committee on Animal Welfare.

### Animals

JR5558 male and female mice were supplied by Charles River (Germany) at 4–5 weeks of age; for the 3-week-old JR5558 mice used for the experiment in Fig. 6, pregnant female JR5558 mice were supplied by Charles River (Germany) and gave birth on-site. For the LCNV experiments, male C57BL/6 mice were ordered from Charles River (France) at 10–12 weeks of age. Animals were allowed to adapt to the new housing conditions for at least 7 days upon arrival. Sample sizes for in vivo experiments were chosen on the basis of previously published experiments with these animal models^47^.

The LCNV mouse model has been described previously^45,59^. In brief, a Phoenix Micron IV retinal imaging microscope (Phoenix Research Labs) coupled to a Meridian Merilas 532α green laser with an intensity of 300 mW (100 ms) was used to place four lesions around the optic nerve of each C57BL/6 mouse eye; the laser injury was located at a distance of 1–2 optic disc diameters from the optic nerve. Mice were 10–12 weeks of age at the time of laser injury. To induce fibrotic lesions of differing severity (Fig. 4), intensities of 150 mW and 500 mW were used in addition to 300 mW.

### In vivo imaging

The CHP probes were designed as previously described^38^. The sCy7.5-CHP was prepared by conjugating the N-terminus with the fluorophore, sCy7.5 (3Helix; excitation/emission: 778/797 nm). For in vivo imaging, mice were injected with sCy7.5-CHPs containing a targeted sequence that binds to collagen (targeted sCy7.5-CHP) or a scrambled sequence as a control (nontargeted sCy7.5-CHP). Because the control sequence contains the same amino acids and sCy7.5 fluorophore, the molecular weight is identical to the targeted sCy7.5-CHP; however, the scrambled sequence prevents binding with denatured or remodeling collagen. sCy7.5-CHPs were injected via the tail vein without anesthesia at a final concentration of 1 nmol per animal (200 μl of 5 μM CHPs in 1× phosphate-buffered saline (PBS)). Mice were anesthetized 5 days after CHP injection by subcutaneous injection of a mixture of fentanyl (0.05 mg/kg), medetomidine (0.5 mg/kg) and midazolam (5 mg/kg). To obtain fundus and cSLO images, eyes were dilated with 1% tropicamide (Théa Pharma). For FA, animals were injected intraperitoneally (10 μl/g bodyweight) with fluorescein (2%; Sigma-Aldrich) and imaged in the FA channel. All images were taken using a Heidelberg Spectralis microscope (Heidelberg Engineering). For the in vivo quantification of sCy7.5-CHP binding to remodeling collagen, the acquired cSLO images were processed using ImageJ^60^ software.

### RPE/choroid flat mount IHC staining

Dissection and flat mount preparation was performed according to a previously published protocol^47^. RPE/choroids were stained in 48-well plates (1 RPE/choroid flat mount per well in 200 μl volume). R-CHPs (2 μM concentration, RED300; 3Helix) in 1× PBS were heated for 5 min at 80 °C and quickly quenched using ice-cold water for 30–60 s before being added to the tissue sections (100 μl per RPE/choroid flat mount). Antibodies diluted in 1× PBS were then added to the RPE/choroid flat mounts and incubated overnight at 4 °C (100 μl per flat mount at 1:100 for a final concentration of 1:200; for a full antibody list, please see Supplementary Table 1). To confirm binding specificity of targeted versus control sCy7.5-CHPs, R-CHPs (2 μM concentration; 3Helix) together with sCy7.5-CHPs (either targeted or control; 3Helix) were added with the primary antibody in 1× PBS without heat-mediated pretreatment. The next day, the flat mounts were washed five times for 5 min with 1× PBS and then incubated for 2 h at room temperature with DyLight-488-conjugated (1:100, #SA-5488; Vector Laboratories) or DyLight-649-conjugated streptavidin (1:100, #SA-5488; Vector Laboratories) and donkey anti-rabbit immunoglobulin G (IgG) (1:200, Alexa Fluor 647, #A31573; Life Technologies) secondary antibody in 1× PBS. RPE/choroid tissue was then washed five times for 5 min with 1× PBS at room temperature before being mounted on Superfrost glass slides using fluorescent mounting medium (#S3032; Dako). Images were acquired with an Olympus VS-ASW scanner equipped with an XM10 camera (Olympus Soft Imaging Solutions software). Specific R-CHP and fibronectin signal areas were selected with the Lasso tool in Adobe Photoshop, and positive areas of R-CHP and fibronectin signal per RPE/choroid flat mount were quantified using ImageJ^60^ software.

### Formalin-fixed, paraffin-embedded IHC staining

Formalin-fixed, paraffin-embedded blocks were cut at 5 μm thickness. Slides were sequentially washed 2 × 3 min with xylene, 100% ethanol, 95% ethanol, 70% ethanol and PBS for deparaffinization. The resulting tissue sections were stained with R-CHPs (1 μM concentration in 1× PBS, RED300; 3Helix) alone or for co-staining experiments, together with rabbit anti-fibronectin antibody (ab23750, 1:200; Abcam). R-CHPs were heated for 5 min at 80 °C and quickly quenched using ice-cold water for 30–60 s before being added to the tissue sections and incubated overnight at 4 °C. The next day, slides were washed with 1× PBS and incubated with 4′,6-diamidino-2-phenylindole (DAPI; 1:1,000, #4083; Cell Signaling Technology) or, for R-CHP and fibronectin co-staining, with DAPI and donkey anti-rabbit IgG (1:200, Alexa Fluor 488, #A21206; Life Technologies) secondary antibody, in PBS for 1 h at room temperature. Tissue was then washed with 1× PBS and mounted using fluorescent mounting medium (#S3032; Dako). Images were acquired with an Olympus VS-ASW scanner equipped with an XM10 camera (Olympus Soft Imaging Solutions software).

### Ang-2/VEGF-A antibody treatment of JR5558 mice

Twenty-one-day-old JR5558 mice received three intraperitoneal injections of anti-Ang-2/VEGF antibody or IgG control (10 mg/kg for both with an injection volume of 10 ml/kg; once weekly on days 21, 28 and 35). At day 37, JR5558 mice were injected i.v. with 1 nmol sCy7.5-CHPs (3Helix) per animal (5 μM in 200 μl 1× PBS); in vivo imaging was performed at day 42 followed by IHC ex vivo. Antibody treatment administration and the qualitative and quantitative analysis of experimental results were made on a blinded basis.

### Statistical analysis

Statistical analysis was performed using GraphPad Prism 8 (GraphPad Software). Unpaired Student’s *t*-test was used to determine statistically significant differences between two groups. For more than two groups, one-way ANOVA followed by Tukey’s multiple comparison test was used. Simple linear regression was used for correlation analyses; *r* and *P* values were calculated using two-tailed correlation with Pearson correlation coefficients.

### Reporting summary

Further information on research design is available in the Nature Portfolio Reporting Summary linked to this article.

## Online content

Any methods, additional references, Nature Portfolio reporting summaries, source data, extended data, supplementary information, acknowledgements, peer review information; details of author contributions and competing interests; and statements of data and code availability are available at 10.1038/s41684-024-01408-0.

### Supplementary information

Supplementary Information Supplementary Table 1 and Figs. 1 and 2.

Reporting Summary

## Data Availability

The data sets generated during and/or analyzed for the reported study are available from the corresponding author on reasonable request.
