# Supplementary material for: In vivo monitoring of active subretinal fibrosis in mice using collagen hybridizing peptides
Source: Lab Anim (NY). 2024 Jul 26;53(8):196–204. doi: 10.1038/s41684-024-01408-0 (PMC11291276; doi:10.1038/s41684-024-01408-0)
Supplement: Supplementary file 1 — Supplementary Table 1 and Figs. 1 and 2. [file 41684_2024_1408_MOESM1_ESM.pdf]

lab animal

https://doi.org/10.1038/s41684-024-01408-0

In vivo monitoring of active subretinal fibrosis in mice using collagen hybridizing peptides

In the format provided by the authors and unedited

Supplementary Tables and Figures

Supplementary Table 1 | Details of the antibodies used in the experiments

Name	Company	Catalog #	Species	Application	Concentration
Collagen I propeptide	Thermo Fisher, Waltham, MA, USA	PA5-35379	Rabbit	Flat mount IHC staining	1:200
Collagen I	Abcam, Cambridge, UK	ab34710	Rabbit	Flat mount IHC staining	1:200
Collagen III	Abcam, Cambridge, UK	ab7778	Rabbit	Flat mount IHC staining	1:200
Fibronectin	Abcam, Cambridge, UK	ab23750	Rabbit	Flat mount and FFPE IHC staining	1:200
Isolectin B4	Sigma, St Gallen, Switzerland	L2140	Biotin conjugate	Flat mount IHC staining	1:200
LOXL2	Abcam, Cambridge, UK	ab96233	Rabbit	Flat mount IHC staining	1:200
Vimentin	Abcam, Cambridge, UK	ab92547	Rabbit	Flat mount IHC staining	1:200

FFPE, formalin-fixed, paraffin-embedded; IHC, immunohistochemistry.

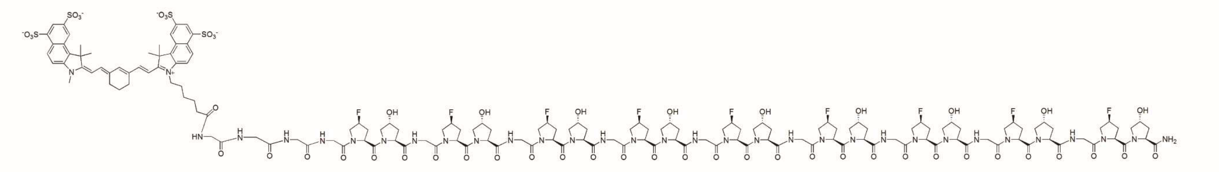

Supplementary Fig. 1 | Chemical structure of a collagen hybridizing peptide labeled with sulfo-cyanine 7.5 for in vivo use

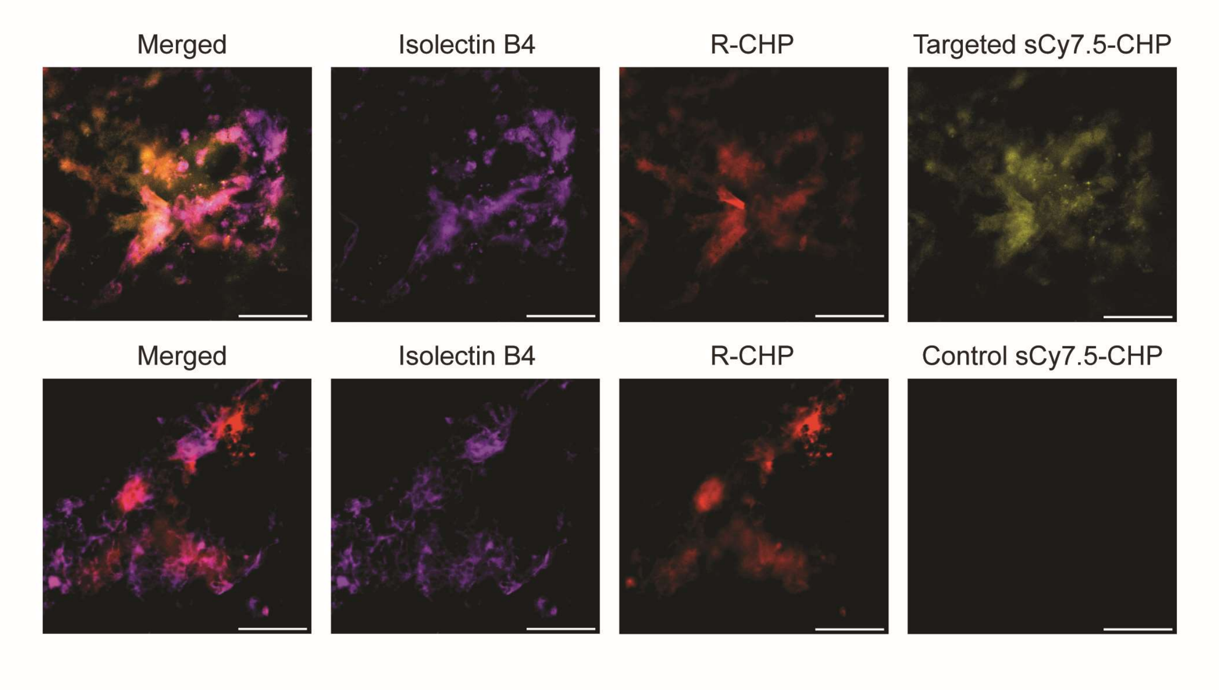

Supplementary Fig. 2 | Immunohistochemistry of sCy3 collagen hybridizing peptide (R-CHP) and sulfo-cyanine 7.5 CHP (sCy7.5-CHP) binding in JR5558 retinal pigment epithelium (RPE)/choroid flat mounts. Representative images of RPE/choroid flat mounts from 84-day-old JR5558 mice stained for R-CHP, targeted and control sCy7.5-CHP binding, and isolectin B4. Scale bars = 50 μm.
